# Western diet shifts immune cell balance

**DOI:** 10.7554/eLife.72787

**Published:** 2021-09-13

**Authors:** Christina M Bergey

**Affiliations:** Department of Genetics, Human Genetics Institute of New Jersey, Rutgers, The State University of New Jersey Piscataway United States

**Keywords:** macaca fascicularis, evolutionary mismatch, diet, monocyte, behavior, inflammation, Other

## Abstract

The immune cells of macaques fed a Western-like diet adopt a pro-inflammatory profile.

**Related research article** Johnson CS, Shively C, Michalson KT, Lea AJ, DeBo RJ, Howard TD, Hawkins GA, Appt SE, Liu Y, McCall CE, Herrington DM, Ip EH, Register TC, Snyder-Mackler N. 2021. Contrasting effects of Western vs Mediterranean diets on monocyte inflammatory gene expression and social behavior in a primate model. *eLife* **10**:e68293. doi: 10.7554/eLife.68293

Our Palaeolithic ancestors would have never experienced anything like a cheeseburger, which has existed for only about 0.1% of human evolutionary history. But they would have loved the processed American cheese, the added bacon, the side of deep-fried potatoes and the 30-ounce soda. Such an energy-dense meal is now readily accessible in high-income countries for the first time in our evolution. Besides this caloric overabundance, numerous nutritional characteristics separate this so-called ‘Western’ diet from what our forebears used to eat. Refined sugars, cereals and vegetable oils, meat from domesticated animals, added sodium, and, in some groups, dairy foods, are now widespread components that would have been largely alien before the advent of farming, animal husbandry and industrialization (Cordain et al., 2005). In the United States, highly manipulated, ‘ultra-processed’ foods which often contain additives like sugar, salt, and fats could now account for two-thirds of the calories that young people consume (Wang et al., 2021).

The modern rise of conditions like obesity, type II diabetes, and cardiovascular disease has led many to posit that an ‘evolutionary mismatch’ is to blame: our physiology evolved in an environment that primarily offered a plant-based diet, and is therefore ill-suited to the modern nutritional landscape (Eaton et al., 1988). Human evolution did not stop with the transition to agriculture, of course, but ten millennia are not enough to fully adapt to such a wholesale dietary transformation. Supporting this conjecture, many chronic diseases are rarer among those who follow the Mediterranean diet, which is thought to be closer to the foods consumed by our ancestral hunter-gatherers due to its abundance of plant-based proteins and relative absence of meat and refined carbohydrates (Mackenbach, 2007). Yet exactly why the Western and Mediterranean diets are associated with such differing rates of disease is unknown.

Now, in eLife, Thomas Register, Noah Snyder-Mackler and colleagues from various institutions across the United States – including Corbin Johnson as first author – report testing a mechanism that may explain the health benefits of the Mediterranean diet over its Western counterpart (Johnson et al., 2021). Their research focused on monocytes, a group of white blood cells which, depending on their exact type, perform distinct immune functions. Based on the genes they express, these cells can either propagate inflammation or halt the process to repair and regenerate tissue (Orekhov et al., 2019).

A prior study, which did not directly investigate diet, found that the monocytes of people with obesity were more likely to be pro-inflammatory (Devêvre et al., 2015). Building on these results, Johnson et al. investigated how diet could influence monocyte activity by feeding macaques a Western- or Mediterranean-like diet.

The change in diet caused a staggering 40% of monocyte genes to be expressed in a different way, with the Western-like diet increasing the activity of genes that regulate pro-inflammatory processes (Figure 1). This result corroborates the findings of a similar study of more limited scope, in which the pro-inflammatory response of monocytes and related cells was dampened in elderly human volunteers who consumed a Mediterranean diet, compared to those on more Western-like foods (Camargo et al., 2012).

**Figure 1.**
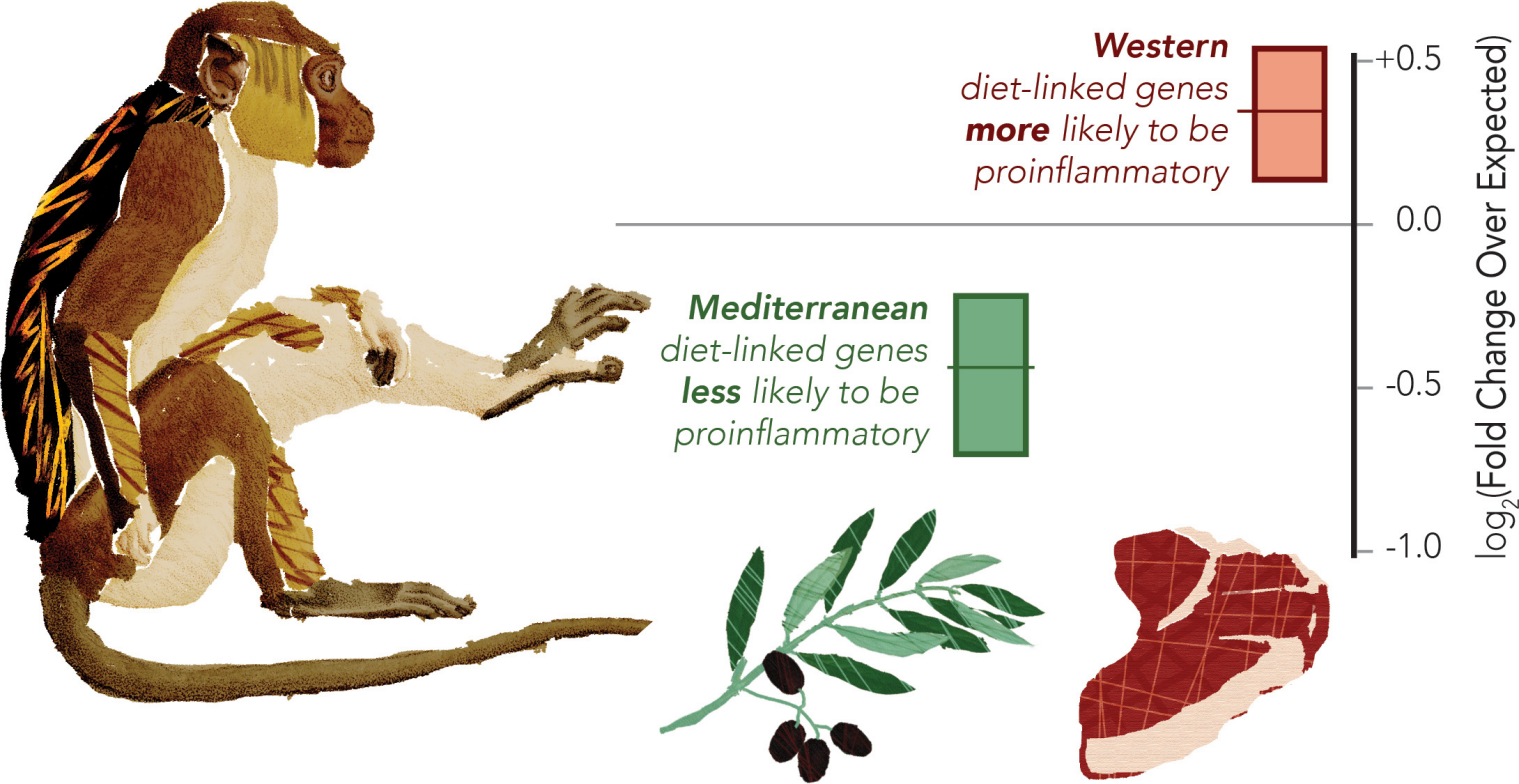
The Western diet shifts monocytes to be pro-inflammatory in a macaque model Macaques were fed either Western or Mediterranean-like diets, and the gene expression of their monocytes was profiled. The proportion of pro-inflammatory genes being activated was then computed (shown in graph). This revealed that genes overexpressed in the Mediterranean-like diet (represented by the olive branch) were less likely than chance (marked as zero) to be pro-inflammatory (data shown in green). Genes that were highly expressed in the monocytes of monkeys fed a Western-like diet (represented by the piece of meat) were more likely than expected by chance to be pro-inflammatory (data shown in red). The gene classification was based on prior direct comparisons of pro-inflammatory and regulatory monocytes (Schmidl et al., 2014).

Image credit: Data adapted from Johnson et al., 2021 (CC BY 4.0).

Macaques are not a perfect analogue to people, but the approach by Johnson et al. bypasses limitations of human studies, which may be limited in time and hampered by participants’ poor recollection of what they had for lunch. Overall, this work bolsters the hypothesis that alterations in monocyte activity explain how the Western diet is associated with inflammation. In turn, these results shed new light on conditions such as cardiovascular disease and cancer, which are also associated with inflammation and cause an immense proportion of modern deaths (Furman et al., 2019).

Several outstanding questions remain: for example, what are the the specific components of the Mediterranean and Western diets that impact monocyte balance and associated diseases? A more nuanced classification of monocyte subtypes could provide additional insight, as would having a clearer idea of the roles human genetic and epigenetic variation play in determining diet response. In addition, genes – in particular those involved in immunity – are connected through delicate networks: could increased inflammation perturb these complex connections, and through this contribute to the evolutionary mismatch between our genomes and our diet? As the Western diet and ultra-processed foods strengthen their hold and now spread across the globe, studies that answer such questions and help to understand the health implications of modern diets are urgently needed.
